# Crystal Structure Elucidation and Anticancer Studies of (-)-Pseudosemiglabrin: A Flavanone Isolated from the Aerial Parts of *Tephrosia apollinea*

**DOI:** 10.1371/journal.pone.0090806

**Published:** 2014-03-07

**Authors:** Loiy Elsir Ahmed Hassan, Mohamed B. Khadeer Ahamed, Aman Shah Abdul Majid, Muhammad Adnan Iqbal, Fouad Saleih R. Al Suede, Rosenani A. Haque, Zhari Ismail, Oon Chern Ein, Amin Malik Shah Abdul Majid

**Affiliations:** 1 EMAN Research and Testing Laboratory, School of Pharmaceutical Sciences, Universiti Sains Malaysia, Penang, Malaysia; 2 Advanced Medical and Dental Institute (IPPT), Universiti Sains Malaysia, Penang, Malaysia; 3 School of Chemical Sciences, Universiti Sains Malaysia, Penang, Malaysia; 4 Institute for Research in Molecular Medicine (INFORMM), Universiti Sains Malaysia, Penang, Malaysia; University of Kentucky College of Medicine, United States of America

## Abstract

*Tephrosia apollinea* is a perennial shrublet widely distributed in Africa and is known to have medicinal properties. The current study describes the bio-assay (cytotoxicity) guided isolation of (-)-pseudosemiglabrin from the aerial parts of *T. apollinea*. The structural and stereochemical features have been described using spectral and x-ray crystallographic techniques. The cytotoxicity of isolated compound was evaluated against nine cancer cell lines. In addition, human fibroblast was used as a model cell line for normal cells. The results showed that (-)-pseudosemiglabrin exhibited dose-dependent antiproliferative effect on most of the tested cancer cell lines. Selectively, the compound showed significant inhibitory effect on the proliferation of leukemia, prostate and breast cancer cell lines. Further studies revealed that, the compound exhibited proapoptotic phenomenon of cytotoxicity. Interestingly, the compound did not display toxicity against the normal human fibroblast. It can be concluded that (-)-pseudosemiglabrin is worthy for further investigation as a potential chemotherapeutic agent.

## Introduction

Chemotherapy can be defined as the use of chemicals to treat cancer by preventing cancer cells from dividing, proliferating and surviving. Chemotherapeutic agents can be divided into two major groups: natural products and synthetic. Currently more than 27% of all prescription drugs are derived from natural sources [1]. This is more significant with regard to anti-cancer drugs in which more than 80% are plant-derived compounds [2]. Synthetic chemotherapy can potentially cause serious side effects, the toxicity associated with the conventional cancer chemotherapy arises primarily from the lack of specificity for tumor cells. Most of the currently available anticancer drugs are designed to have selective toxicity towards rapidly dividing cells [3]. It is reported that natural compound(s) especially of plant origin, with selective cytotoxicity against specific tumor cell line, can offer either prophylaxis (chemoprevention) or a relatively safer treatment option with minimum side effect [4]. Chemopreventive agents derived from natural products or their synthetic derivatives have shown cell growth inhibition, antiproliferation and apoptosis in various cancer cell lines [5]. For example, retinoids and antiestrogens are known to block or delay the progression of transformed cells by modulating cell proliferation or differentiation, these agents are believed to promote cytostatic effects [6], [7], [8].

*Tephrosia apollinea* is a member of family Leguminosae and genus Tephrosia. *T. apollinea* is a perennial shrublet which is widely distributed in Africa. In Sudan the shrublet is abundantly distributed in the Nile Valley, in the sub-Saharan northern region and along the coast of the Red Sea. Several reports have indicated that the extract from some species of the genus Tephrosia possess dynamic pharmacological activities such as, piscicidal, insecticidal and anti-cancer properties [9]. Furthermore, a review of the literature indicates that a number of species of Tephrosia have been studied for their chemical compositions [10]. Studies analyzing their chemical composition revealed the presence of rotenoids, isolflavones, flavanones, chalcones, and flavones. The study by Abou-Douh et al. [11] reported the presence of complex prenylated flavones derived from 7-oxygenated compounds in the extracts of *T. apollinea*. The most recent work on *T. apollinea* describes the isolation of stereoisomers, (-)-semiglabrin and (-)-pseudosemiglabrin [8]. Additionally, the study also explored the stereochemistry of (-)-semiglabrin using x-ray crystallography [11]. Using an *in vitro* model of anticarcinogenesis, the study [11] reported that, (-)-pseudosemiglabrin showed no significant anticarcinogenic activity in a cell and enzyme based *in vitro* assay against H4IIE rat hepatoma cells. The study [8] reported that (-)-pseudosemiglabrin failed to inhibit the enzymes (cytochrome 1A and quinone reductase) involved in carcinogen metabolism and detoxification. The study [8] further reported that the compound did not show inhibitory effect on the enzymes (cyclooxygenase-1 and cyclooxygenase-2) actively involved in tumor-promoting mechanism.

In the present study, extracts of the aerial parts of *T. apollinea* were subjected to bioassay-guided fractionations, which resulted in isolation of (-)-pseudosemiglabrin (SSG). The structural and stereochemical features were confirmed by spectral and X-ray crystallographic techniques. The compound was evaluated for its potential antiproliferative effect against a panel of human cancer and normal cell lines. Furthermore, an attempt was made to understand the mode of cytotoxicity induced by SSG in cancer cells by performing Hoechst 33342 and rhodamine 123 fluorescence assays.

## Results and Discussion

### Plant Extract and Isolation of Active Compound

**Figure 1:**
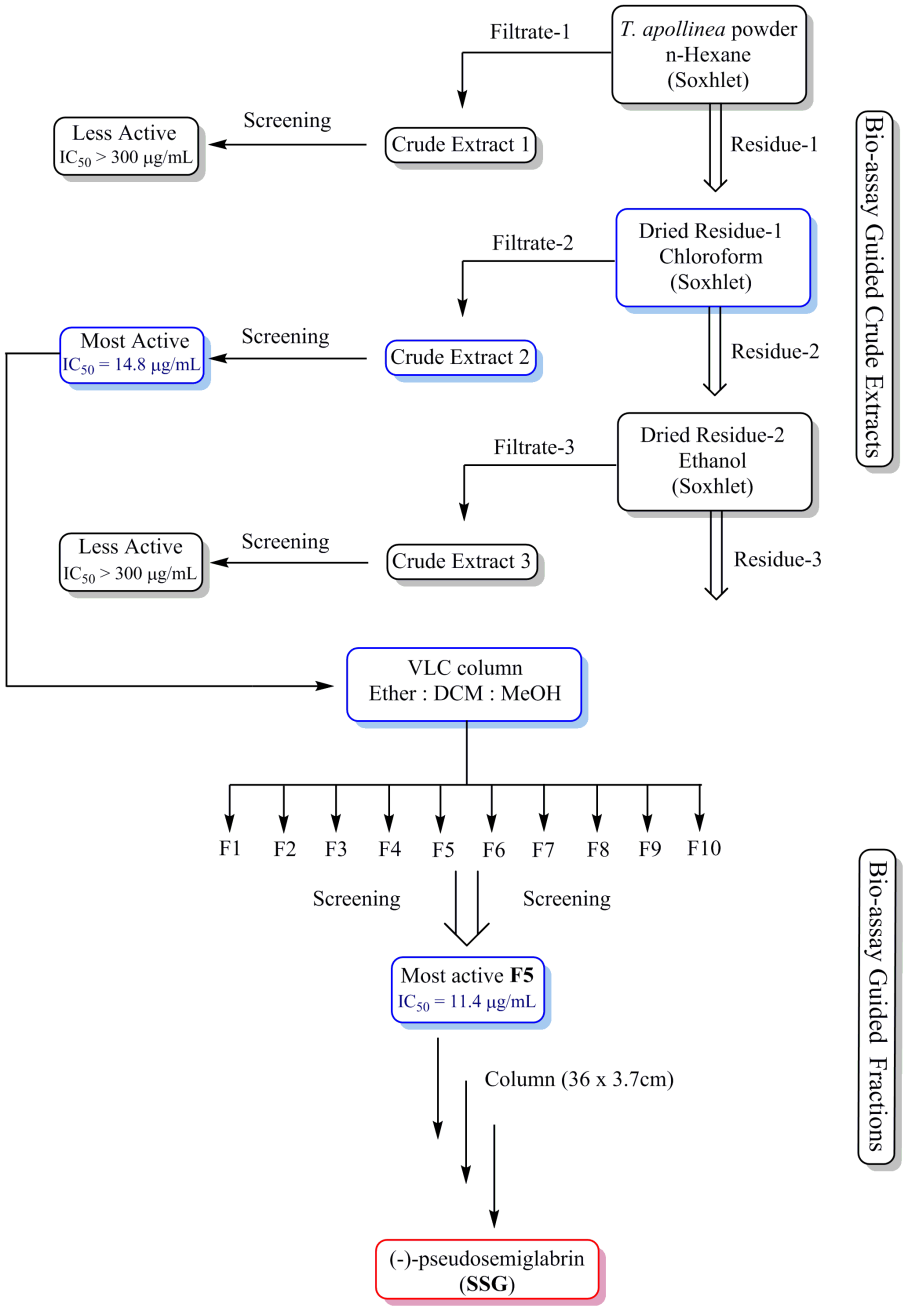
Isolation of (-)-pseudosemiglabrin (SSG). Schematic diagram showing bio-assay guided sequential extraction and fractionation of *T. apollinea* leading to separation of SSG.

Aerial parts of *T. apollinea* were sequentially extracted with n-hexane, chloroform and ethanol to obtain three respective crude extracts (Figure 1). Among all the extracts, chloroform extract showed most potent anti-proliferation activity against HL-60 (IC_50_ 19.2 µg/mL), K562 (14.8 µg/mL) and MCF-7 (16.4 µg/mL) cell lines. Chromatographic fractionation of chloroform extract yielded ten fractions (F1-F10). Among all the fractions, F5 was found to be the most active fraction against the proliferation of HL-60 (IC_50_ 13.6 µg/mL), K562 (26.1 µg/mL) and MCF-7 (11.4 µg/mL). Thus, F5 was further chromatographed using gradient elution of n-hexane-dichloromethane to yield SSG. A detailed procedure is described in the experimental part.

### Spectroscopy

**Figure 2:**
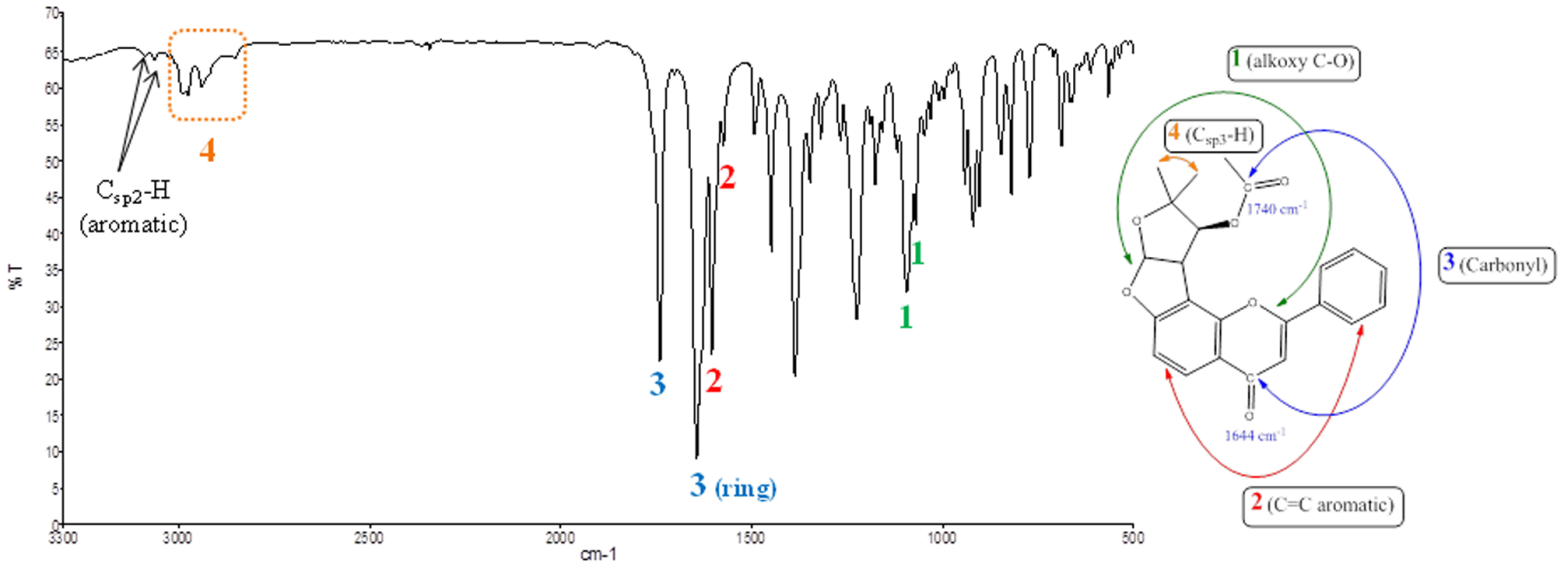
FT-IR spectral features of (-)-pseudosemiglabrin. The figure highlights alkoxy (1), C = C aromatic (2), carbonyl (3), and alkyl group (4) stretches of SSG.

SSG was obtained as light green crystalline plates, M.P: 170–180°C. The molecular mass was determined by liquid chromatography-mass spectroscopy (LC-MS) and showed a molecular ion peak at 393.11. The ultraviolet (UV) spectrum showed absorption at λ max 306, 256, (sh) and 215 nm indicating the flavone characteristics of the compound SSG [11], [12], [13]. The infrared (FT-IR) spectrum showed a strong and sharp vibrational band at 1740 cm^−1^ that indicates the presence of carbonyl (CO) moiety, more likely CO of an acetate group [14]. Also, a medium intensity band at 1640 cm^−1^ attributed the CO of pyranone ring [15], [16]. Furthermore, a vibrational band at 1574 and 1604 cm^−1^ ascribed the benzene ring carbon-carbon stretch. These prominent characteristic features indicate the presence of flavones, a class of compounds based on a backbone of 2-phenylchromen-4-one [11], [17], [18], [19]. The presence of alkyl groups was imputed by two vibrational bands at 2850, 2939 and 2974 cm^−1^ [20], [21]. These three weak bands indicate the presence of alkyl groups attached to flavone backbone (Figure 2).

Further, the title compound was characterized by ^1^H and ^13^C-NMR. The ^13^C DEPT-135 and 145 NMR spectra recorded in CDCl_3_ at 125.7 MHz at room temperature are shown in Figures S1 and S2, respectively in File S1. The 2D HSQC and HMBC NMR spectra of SSG are shown in Figures S3 and S4, respectively. Figures S5 and S6 in File S1 illustrate the characteristic diagonal component and cross peaks of SSG obtained in 2D TOCSY and COSY NMR spectra, respectively. The data obtained from these spectral analyses were found to be comparable with that of the previous reports [11], [22].

### Crystallography

Crystals of title compound suitable for x-ray crystallographic study were obtained by slow evaporation of the compound in dichloromethane/n-hexane solvent system (1∶3). The crystals appeared as light green plates.

**Figure 3:**
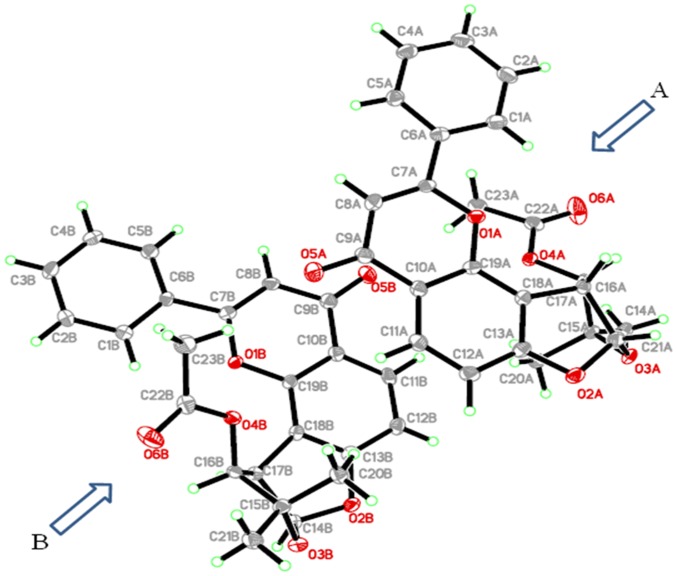
ORTEP (Oak Ridge Thermal Ellipsoid plot) picture of (-)-pseudosemiglabrin with the displacement ellipsoid drawn at 50% probability. The figure presents two crystallographically different molecules (A and B) of title compound.

The compound crystallized in orthorhombic space group *P*2_1_2_1_2_1_, with two crystallographically different molecules having slightly different geometric parameters. Each unit of the compound consists of two benzene, one pyranone and two tetrahydrofuran (THF) rings. In addition, an acetate group is attached with one of the THF groups. The crystal refinement data is shown in Table 1, whereas the selected bond lengths and angles of both the crystallographically different units, A and B are listed in Table S1 and Table S2, respectively in File S1. The detailed aspects of the crystal structure are illustrated in Figure 3.

**Table 1:** Crystal Data and Structure Refinement Details for (-)-Pseudosemiglabrin.

Parameters	Crystal Data
Formula	C_23_H_20_O_6_
Formula weight	392.39
Crystal system	Orthorhombic
Space group	*P*2_1_2_1_2_1_ (No. 19)
Unit cell dimensionsa (Å)	9.4533(2)
b (Å)	9.7884(2)
c (Å)	40.0762(8)
V (Å^3^)	3708.36(13)
*Z*	8
Density (calcd) (gm/cm^3^)	1.406
Abs coeff (mm^−1^)	0.102
F(000)	1648
Crystal size (mm)	0.12×0.18×0.44
Temperature (K)	100
Radiation (Å)	MoKa 0.71073
*θ* Min, max (°)	2.0, 30.1
Dataset	−11: 13 ; −13: 13 ; −42: 56
Tot.; Uniq. Data	36701; 10853
R (int)	0.058
Nref, Npar	10853, 529
R, wR_2_, S	0.0570, 0.1317, 1.01

S  =  Goodness of fit, in general value of S should be close to 1.

*w*R2  =  Weighted Residual Factor is the most closely related to the refinement against squared structure factors. The weighting factor *w* is indivisually derived from the standard uncertanities of the measured reflections and expresses the confidence we have in every single reflection. This factor should be minimum during refinement

R  =  Unweighted residual factor. This factor should also be minimum during refinement.

Nref  =  Number of independent reflections

Npar  =  the number of refined parameters

R_int_  =  The merging residual value, reflects the summations involve all the input reflections for which more than one symmetry equivalent are averaged.

**Figure 4:**
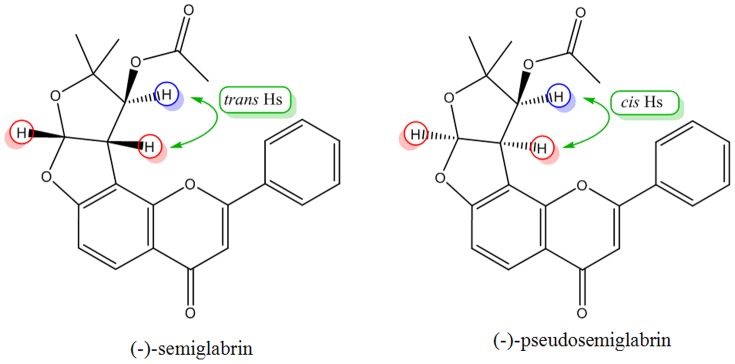
Stereochemical structures of (-)-semiglabrin and SSG.

It is an interesting and rarely observed phenomenon [23], [24] in crystallography when the molecules of the same compound pack with slightly different geometries. This phenomenon, however, might not alter the biological properties of the compound. Nevertheless, the stereoisomers have some significant differences regarding physical properties like melting point, crystal packing, crystal color etc [25], [26], [27] as well as biological efficacies [26], [27]. For example, the crystal structure of (-)-semiglabrin, a diasteroisomer of title compound (Figure 4) has been reported by Abou-Douh et al [11] where the single crystals appeared as colorless needles instead of light green plates for SSG in the current report.

The melting point of the crystals was about 258–260°C for needles whereas in the current study the observed melting points were in the range of 168–170°C. Also, a number of bond angles are slightly different (1.5±0.5°) than these angles in its stereoisomer, (-)-semiglabrin. For example, O4A-C22A-C23A in SSG is 110.29(16)° whereas in (-)-semiglabrin the same bond angle is oriented at 108.10(11)°. The other bond angles are C16A-O4A-C22A and C15A-O3A-C14A having angles 117.41(15)° and 110.58(14)° in the current structure whereas in its isomer these are 118.8(10)° and 111.10(9)°, respectively (Refer to Table S1 in File S1). Furthermore, these crystallographically different units also have difference in bond angles compared to each other e.g., C21A-C15A-C20A and C15A-O3A-C14A in structure A have a difference of ±0.5° compared to the same angles in structure B (Figure 3). The variations in the bond angles and bond lengths between the two units are given in Tables S1 and S2 in File S1.

Moreover, in both the geometrically different molecules, the THF rings make the dihedral angles of 110±0.5° and 116±0.5° with each other at both ends. All the three rings (two benzene and one pyranone) are in the same plane with a benzene and pyranone ring connected through a single bond. Such bonding situations have been more likely found to have some orientations in horizontal plane [28], [29], [30], [31], [32] however, in the current case both the rings are in the same plane. This might be due to packing effects where the molecules have π-π stacking interactions that might keep these rings in the plane. Figure S7 in File S1 illustrates the crystal packing pattern, which shows that the molecules are connected through the C = O - - - H bonding in a three dimensional network.

### Effect of SSG on Proliferation of Cancer Cell Lines

**Figure 5:**
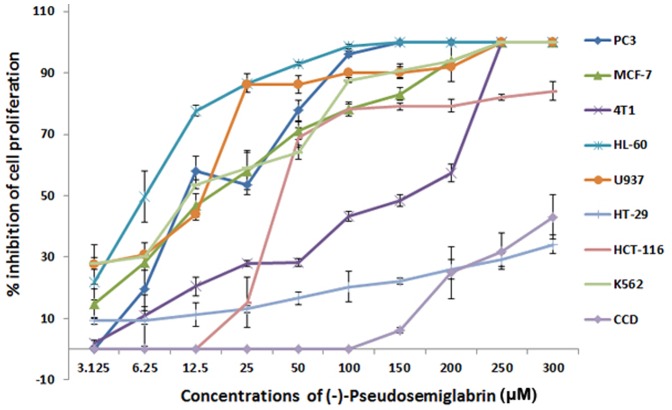
Dose-dependent anti-proliferative effect of SSG on, HCT 116, MCF-7, PC3, HL-60, K562, 4T1, HT-29, U937 and CCD cell lines was assessed by MTT-assay. (Values are represented as mean ± SD, n = 3).

Antiproliferative effect of SSG was tested against nine tumor cell lines and one normal cell line using MTT assay. The median inhibitory concentration (IC_50_) values were calculated for each cell line [33] and the values are given in Table 2. The compound showed selective cytotoxicity against six cancer cell lines namely MCF-7 (breast cancer, IC_50_ 18.24 µM), PC3 (human prostate cancer, IC_50_ 6.11 µM), HL-60 (human promyelocytic leukemia, IC_50_ 15.7 µM), K562 (human immortalized myelogenous leukemia, IC_50_ 22.3 µM), U937 (human histiocytic leukemia, IC_50_ 5.76 µM) and HCT-116 (human colorectal tumor, IC_50_ 19.6 µM) cells. Whereas, the compound exhibits either moderate or poor cytotoxic effects against HT-29 and HepG2 with IC_50_ values 135.12 and > 300 µM, respectively. In addition, the compound did not show considerable toxicity against normal cell line i.e. CCD-18Co (IC_50_  =  327.25 µM). The results were compared with the respective standard reference drugs, tamoxifen, betulinic acid, 5-fluorouracil and imatinib. Figure 5 shows the graphical illustration of the dose-dependent antiproliferative effect of SSG on various human cancer cell lines. Apparently this finding is in contrast with that of the previous study in which SSG was reported to be inactive in a cell-enzyme based *in vitro* assay conducted using H4IIE rat hepatoma cells, as the compound failed to show inhibitory effects on the initiation, promotion, and progression stages of the assay [11]. Nevertheless, the results of the present study agree in part with the previous findings in the sense that SSG did not show anti-proliferation activity against human hepatic carcinoma (HepG2) cells as the IC_50_ value was found to be more than 300 µM. Interestingly, the compound displayed selective cytotoxicity against prostate, breast, leukemia and colon carcinoma cell lines.

**Table 2:** IC_50_ Values of (-)-Pseudosemiglabrin (SSG) on Various Human Cancer Cell Lines^a^.

Test samples^n^		Cell line (IC_50_ in µM)								
	HCT-116	HT-29	MCF-7	PC3	HL-60	K562	U937	4T1	HepG2	CCD
**SSG**	19.6	135.4	18.2	6.1	15.7	22.3	5.76	148.7	>300	327.2
**Tamoxifen**	-	-	7.8	-	1.6	-	-	16.3	-	-
**Betulinic Acid**	-	-	-	21.3	-	-	-	-	-	145
**5-fluorouracil**	4.7	9.3	-	-	-	-	-	-	4.2	-
**Imatinib**	-	-	-	-	-	0.2	-	-	-	-

a The median inhibitory concentrations (IC_50_) were determined by nonlinear regression analysis of log-concentration-response curves of 3 different tests (n = 3).

**Figure 6:**
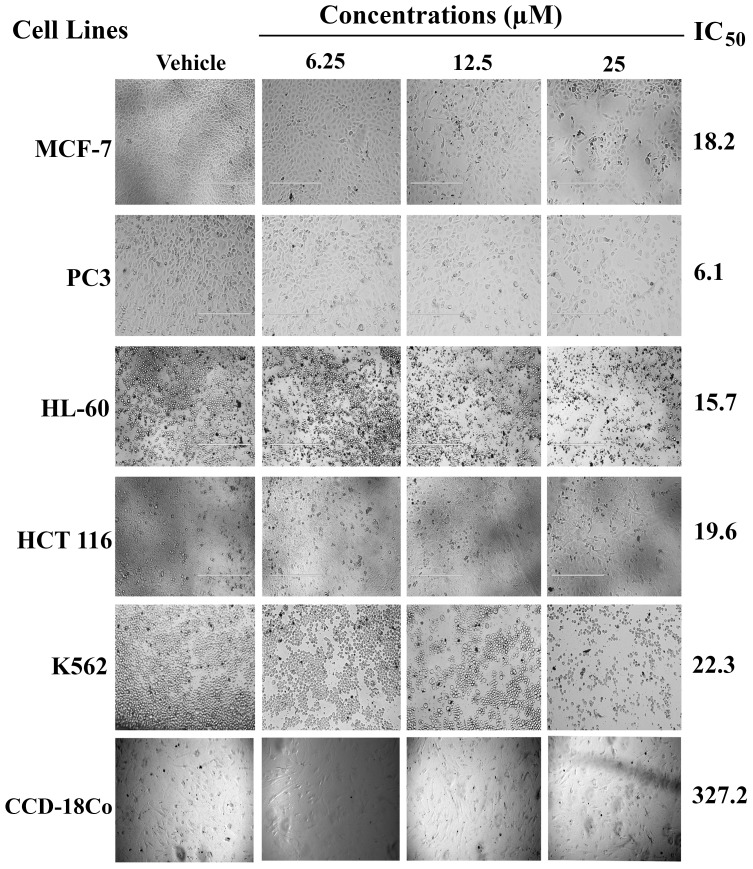
Photomicrographic images of cancer cell lines, taken under an inverted phase-contrast microscope at × 200 magnification using a digital camera at 48 hours after treatment with the SSG.

Figure 6 shows the effect of SSG on various human cancer cell lines. The results revealed that SSG was nontoxic to normal colonic fibroblast (CCD-18Co) cells (IC_50_  =  327.25 µM).

A study reports that γ-Pyranone derivatives isolated from *Erigeron annuus* showed poor antiproliferative activity against human hepatoma (SMMC-7721), embryo liver (L-02) and leukaemia (HL-60) cell lines [34]. However, the study revealed that a γ-Pyranone derivative with ester group attached to the long side chain exhibited improved cytotoxicity especially against HL-60. Similarly, SSG also contains an ester group attached to one of the tetrahydrofuran rings and showed a promising cytotoxicity against HL-60 (IC_50_  =  15.7 µM). A number of studies have shown that compounds containing ester groups induce anticancer activity against cancer cells [35], [36], [37], [38] especially against HL-60 cell line [39].

Furthermore, Kajimoto and coworkers isolated Sophoranone, a flavone, from a traditional Chinese medicine, namely Shan Dou Gen that induced apoptosis in human leukemia (U937) cells via formation of reactive oxygen species and opening of mitochondrial permeability transition pores (IC_50_  =  21.5 µg/mL) [40]. In the current study, the tested compound showed even better cytotoxic effect with IC_50_  =  5.76 µM. Moreover, comparing with some other flavones tested against U937 cell line [41], [42], [43], results of the present study revealed that SSG exhibited more pronounced cytotoxic efficacy. According to best of our knowledge this is the first report that describes anticancer potential of SSG against a panel of cancer cell lines, where it significantly showed selective anti-proliferative activity against leukemia, prostate, breast and colon cancer cell lines.

### SSG Induces Morphological Modifications and Nuclear Condensation in the Cancer Cells

During apoptosis, cell exhibits a series of characteristic morphological and biochemical events, such as nuclear condensation, DNA fragmentation, dissolution of chromatin, and alterations in cellular membrane [44]. To detect particular events of apoptosis and trace mechanisms responsible for the apoptotic cell death, we employed commonly used staining assays to detect changes in the nucleus and mitochondria of treated cells.

**Figure 7:**
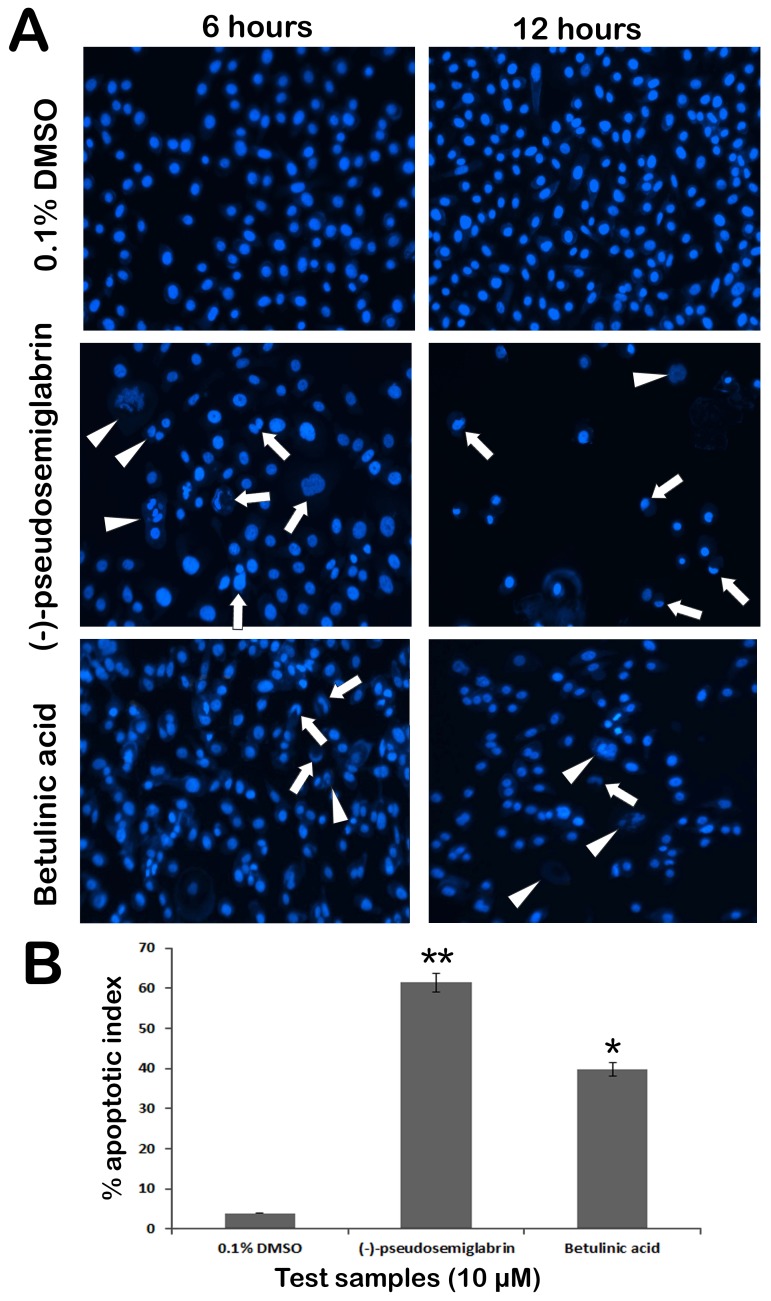
A) The photomicrographs depict the images of PC3 cells with Hoechst 33258 stain taken at 6 and 12 hours after treatment. The cells treated with SSG revealed clear signs of proapoptosis. The cells treated with 0.1% DMSO (Vehicle) showed prompt and evenly distributed nucleus with fully extended pseudopodial like projections of cell membrane. Whereas, the cells treated with SSG (10 µM) displayed blebbing of cellular membrane and the typical apoptotic changes in the chromatin structure. The arrows indicate the clear signs of nuclear condensation including the half moon (crescent) shaped apoptotic nuclei. The arrowheads indicate the chromatin dissolution, breakdown and fragmentation. The standard reference, betulinic acid also showed the similar induction of apoptosis in the cells. B) Graphical representation of percentage of apoptotic indices. The apoptotic index for each test group was expressed as a percentage of the ratio of apoptotic cells number to the total cell number in 10 different fields. Values are presented as mean ± SD (*n* = 10), * represents p<0.05 and ** represents p<0.01.

**Figure 8:**
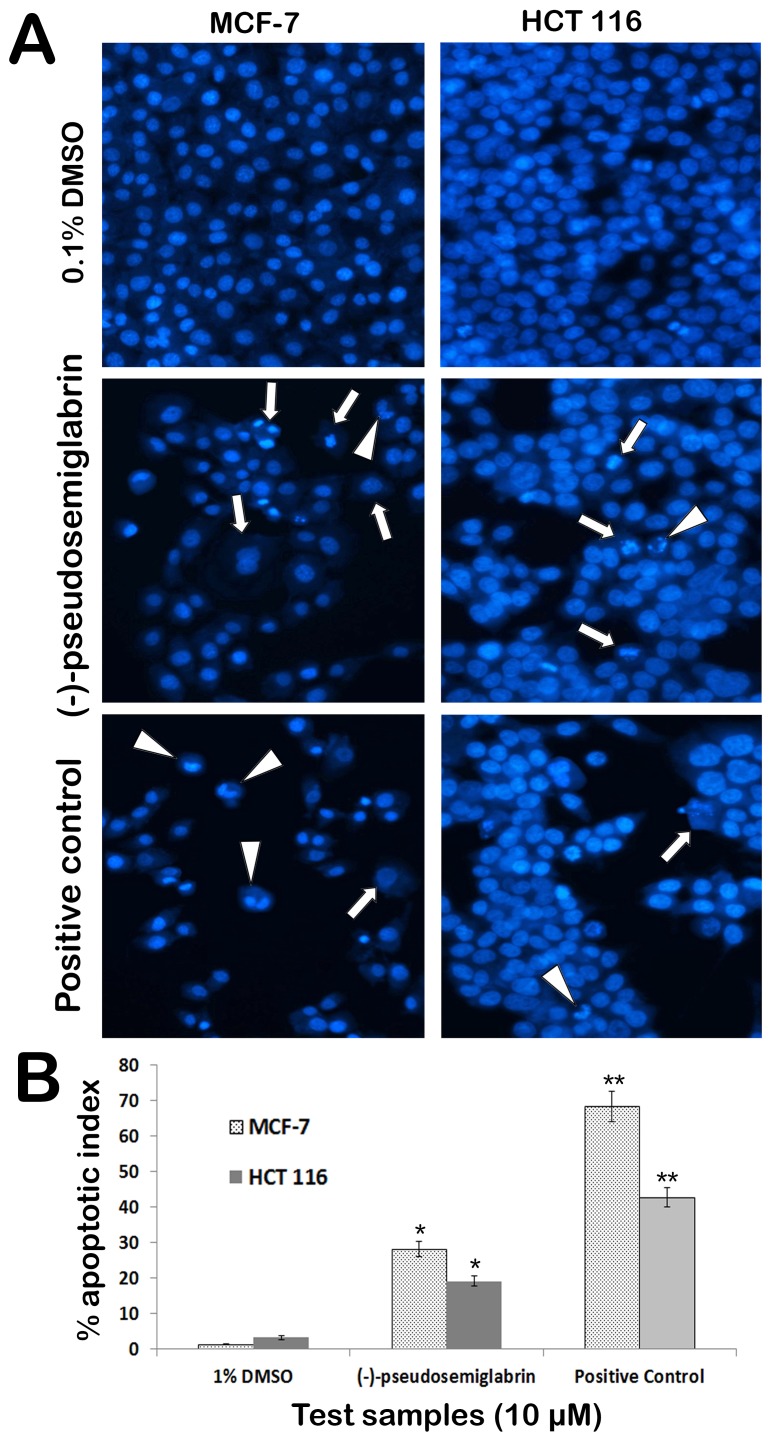
A) The photomicrographs depict the images of MCF-7 and HCT 116 cells with Hoechst 33258 stain. The cells from negative control (0.1% DMSO) demonstrated lively growing cells with prominent nuclear and other cellular features. The cells treated with SSG exhibited obvious characteristic changes of apoptosis. The arrowheads indicate the condensed, fragmented and crescent shaped nuclei. The arrows indicate the chromatin dissolution, breakdown and fragmentation. The results can be compared to that of the standard reference, betulinic acid. **B**) Graphical representation of percentage of apoptotic indices for different test groups. The apoptotic index (%) induced by SSG in MCF-7 and HCT 116 cells was 28.3±2.1 and 19.2±1.4%, respectively. Tamoxifen was used as the positive control for MCF-7 cells which caused significant (68.4±4.3%, p<0.01) apoptotic changes in the cells. 5-fluorouracil was used as the positive control for HCT 116 cell, which caused 42.8±2.6% of apoptotic index. Values are presented as mean ± SD (*n* = 10), * represents p<0.05 and ** represents p<0.01.

In the present study, adherent cells (PC3, MCF 7 and HCT 116) were selected to study morphological modifications and nuclear condensation using Hoechst 33342 stain. The typical apoptotic morphological changes were observed in the cells treated with SSG in a time dependent manner (Figure 7A). However, untreated cells displayed prominent nuclei and intact cell membrane without significant changes in cellular morphology. After 6 hr of SSG (10 µM) treatment, the nuclei started to condense and the chromatin distributed irregularly throughout the cytoplasm. The cells revealed shrunken, crescent-shaped nuclei with condensed chromatin, which are the sings of early stage of apoptosis (Figure 7A). After 12 hr treatment, several cells showed the discrete chromatin bodies which are the characteristic sign of karyorrhexis, a later stage of apoptosis. The apoptotic index (Figure 7B) for untreated PC3 was 3.9±0.07%, for, MCF-7 the apoptotic index calculated was 1.35±0.08% and that of HCT 116 cells was 3.3±0.6% which were increased to 61.4±2.3% (PC3), 28.3±2.1% (MCF-7) and 19.2±1.4% (HCT 116) following treatment with SSG for 12 hr (Figures 8A and 8B).

### SSG Reduces Mitochondrial Membrane Potential in PC3 Cells

**Figure 9:**
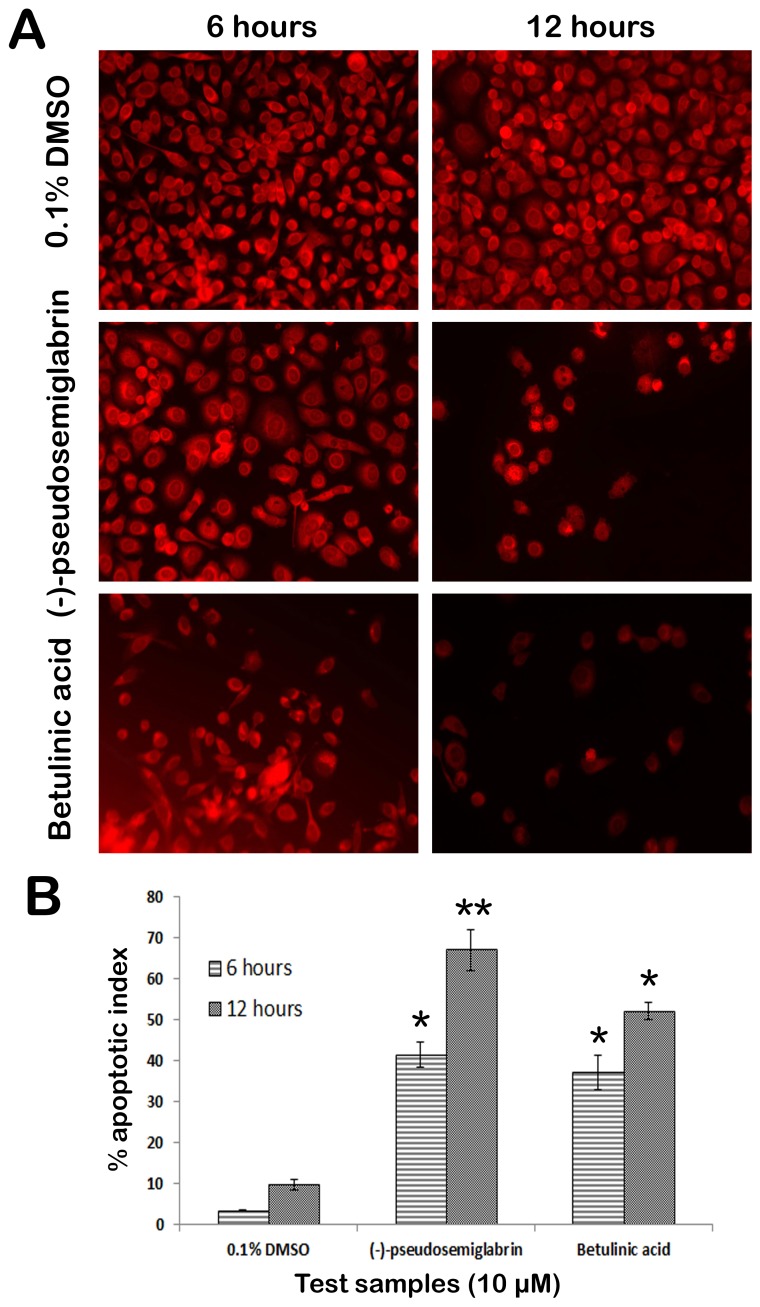
A) The photomicrographs illustrate the ability of SSG to disrupt the mitochondrial membrane potential. The mitochondrial membrane potential in PC3 cells was evaluated by visualizing the uptake of the lipophilic cation dye rhodamine into mitochondria. The results showed that the rhodamine 123 fluorescence signal decreased substantially with respect to the decrease of mitochondrial membrane potential due to the treatment with SSG. **B**) Graphical representation of percentage of apoptotic indices. The apoptotic index for each test group was expressed as a percentage of the ratio of unstained cells number to the total cell number in 10 different fields. Values are presented as mean ± SD (*n* = 10), * represents p<0.05 and ** represents p<0.01.

Rhodamin 123 is a cationic probe which can be readily absorbed and accumulated in mitochondria of a live cell [45]. A loss of mitochondrial membrane potential (ΔΨ) is a marked indication of early apoptotic events [46]. To investigate whether the apoptosis induced by SSG in PC3 cells involved the loss of mitochondrial integrity, the mitochondrial membrane potential in PC3 cells was evaluated by visualizing the uptake of the lipophilic cation dye rhodamine 123 by mitochondria. The treated and untreated cells were exposed to rhodamine 123 and the intensity of rhodamine in the cells was observed (Figure 9A). When the mitochondrial membrane potential decreases, the rhodamine 123 uptake by the cells also decreases and consequently the florescent signal reduces exponentially. Results of the present study showed an obvious intensification of fluorescence in the untreated cells, whereas the signal significantly reduced in the cells treated with SSG (10 µM), which suggests the loss in mitochondrial membrane potential. Furthermore, the fluorescent intensity decreased with increasing the treatment duration (Figure 9A). The apoptotic indices after 6 and 12 h treatment with SSG were 41.4±3.1 and 67.1±4.2%, respectively (Figure 9B). This result reveals that, there is a remarkable reduction in the mitochondrial membrane potential of PC3 cell line could be due to the induction of apoptosis caused by SSG.

Flavonoids and their metabolic precursors such as flavanones and phenolics have shown to possess promising anticancer properties. Our previous study [47] reported that plant flavonoids particularly polymethoxylated flavonoids, such as rosmarinic acid, eupatorin, sinensetin, and 3′-hydroxy-5,6,7,4′- tetramethoxyflavone exhibit anticarcinogenic properties *via* suppressing oxidative stress in the cells. However, in the present study the photomicrographs of the affected cells in Hoechst 33342 and rhodamin 123 staining assays revealed that the toxicity caused by SSG could probably due to the induction of apoptosis pathway, as the affected cells clearly showed the unique features of apoptosis such as membrane blebbing, nuclear condensation and apoptotic bodies in cytoplasm of the cells. Few cells also showed the crescent shaped nuclei which indicate the advanced stage of apoptosis.

Chemopreventive agents from natural products that inhibit the transformation of normal cells to premalignant cells or the progression of premalignant cells to malignant cells are believed to function by modulating processes associated with xenobiotic biotransformation, with the protection of cellular elements from oxidative damage, or with the promotion of a more differentiated phenotype in target cells. Nevertheless, there is an increasing number of chemopreventive agents (e.g., retinoids, nonsteroidal anti-inflammatory drugs, polyphenols, flavonoids and vanilloids) which demonstrate apoptosis in premalignant and malignant cells *in vitro* or *in vivo* [5], [48], [49].

## Experimental Section

### Materials and Methods

#### Chemicals and Reagents

Cell culture reagents were purchased from Gibco, USA; RPMI 1640 medium; catalogue number (A10491-01), Dulbecco's Modified Eagle Medium; Catalogue number (31100-035), Trypsin and heat inactivated foetal bovine serum (HIFBS) were obtained from GIBCO, UK. MTT (3-(4,5-Dimethylthiazol-2-yl)- 2,5diphenyl tetrazolium bromide) was purchased from Sigma-Aldrich, Germany. Dimethyl sulfoxide (DMSO) was purchased from Fluka, USA. F-12K medium catalog number (30-2004) form ATCC, USA.

The isolated SSG was dissolved in DMSO to obtain 10 mg/mL stock solution and stored at 4°C. For treatment, SSG was diluted in indicated cultured medium at the indicated concentrations in each experiment.

#### Plant Material

The aerial parts of *T. apollinea* were collected from the Botanical garden of National Center for Research, Khartoum, Sudan in the year 2012 with the institutional permission. Taxonomical authentication was done by senior taxonomist, Mr. Wail Alsadiq and the voucher specimen (Ref. No. 200784/2012) is deposited at the herbarium of the Institute of Medicinal and Aromatic Plant, National Centre for Research, Sudan. The present work does not involve experimentation on animals, therefore, no permissions were required from animal ethics committee.

### Extraction and Fractionation

The plant materials were dried at room temperature and ground to a fine powder. Initially, 50 g of material was extracted with 200 mL n-hexane overnight. The filtrate was collected and the residue was brought to dryness and extracted with 200 mL chloroform following the same methods as for n-hexane. The filtrate was collected and the residue was brought to dryness and extracted with ethanol following the same method as for n-hexane and chloroform. Chloroform extract was found to be more active against cancer cell lines (HL-60, K562 and MCF-7), thus it was subjected to fractionation. For large scale extraction, 400 g of the plant material was extracted with chloroform using Soxhlet apparatus.

### Isolation of (-)-Pseudosemiglabrin (SSG)

Chloroform extract (7 g) of *T. apollinea* was subjected to vacuum liquid chromatography which was performed on column (10×7 cm) packed with silica gel of particle size (0.04–0.06 mm, 60–120 mesh). Solvent mixtures of petroleum ether, dichloromethane, and methanol were used in sequence of increasing polarities. Eluents of 100 mL each were collected and monitored by thin layer chromatography (TLC). The eluents with similar TLC chromatogram pattern were pooled together to obtain totally ten fractions. Bioassay results showed that fraction 5 (1.3 g) was the most active against the cell proliferation, which was further applied to column (36×3.5 cm) packed with silica gel (65 g) of particle size 0.063–0.200 mm by gradient elution, starting with hexane–chloroform combination (95∶05), followed by increasing amount of chloroform and methanol. A total of 124 fractions (10 mL each) were collected. Fractions 20–93 were combined together and purified using pen column (16×2 cm) by gradient elution starting with petroleum ether–dichloromethane mixture (90∶10) followed by increasing amount of dichloromethane. A total of 60 fractions (10 mL) were collected. Fractions 11–52 were combined on the basis of TLC profile to give one spot (600 mg). TLC analysis of the substance in several different solvent systems revealed a single fluorescent spot under UV light, with faint red spot of chlorophyll. Subsequently, the SSG was purified from the chlorophyll by fractional crystallization in mixture of n-hexane and dichloromethane to produce light green crystals (400 mg).

### Characterization Techniques

The purified crystalline material was characterized by FT-IR spectrophotometer (FTIR-2000, Perkin Elmer, USA) using potassium bromide (KBr) disc method. According to this method sample was mixed with oven dried IR grade KBr and ground to fine powder. A disc (12.7 mm diameter and around 1 mm thickness) was obtained using hydraulic press (capacity 15 tons max.) at 8 tons for about half minute. The spectrum was scanned at infrared region of 400–4000 cm^−1^.

Furthermore, the purified compound was analyzed by FT-NMR spectrometer (Bruker 500 MHz) in deuterated chloroform (CDCl_3_). The NMR peaks were labeled as singlet (s), doublet (d), triplet (t), and multiplet (m), chemical shifts were referenced with respect to solvent signals.

The molecular weight of the compound was determined by liquid chromatography-mass spectrometer (LC-MS). The sample was prepared in HPLC grade methanol (10 mg/ml) and was filtered through 0.45 micron filter.

The single crystals obtained were analysed by Bruker SMART APEX2 CCD area-detector diffractometer. The molecular graphics were constructed by Bruker SHELXTL software.

#### Characteristic Patterns of SSG

Light green crystals, m. p. 174°C. ^1^H NMR (500 MHz, CDCl_3_) δ ppm: 1.17 (s, 3H, 1 × CH_3_), 1.41 (s, 3H, 1 × CH_3_), 1.51 (s, 3H, 1 × CH_3_), 4.65 (d. d, 1H, 1 × CH, ^3^ *J*_H.H_  =  9.0 Hz & ^3^ *J*_H.H_  =  6.5 Hz), 5.61 (d, 1H, 1 × CH, ^3^ *J*_H.H_  =  9.0 Hz), 6.54 (d, 1H, 1 × CH, ^3^ *J*_H.H_  =  6.5 Hz), 6.78 (s, 1H, Ar-H), 6.98 (d, 1H, Ar-H, ^3^ *J*_H.H_  =  8.5 Hz), 7.58 (m, 3H, Ar-H), 7.86 (d.d, 2H, Ar-H, *J*  =  7.7 Hz & *J*  =  2.0 Hz), 8.20 (d, 1H, Ar-H, ^3^ *J*_H.H_  =  8.5 Hz). ^13^C {^1^H} NMR (125.5 MHz, CD_3_Cl) δ ppm: 20.3, 23.2, 27.6 (3 × CH_3_), 47.9, 84.6, 107.6, 108.9, 111.4, 111.7, 126.2, 128.7, 129.1, 131.4, 131.7, 153.8, 162.6, 164.5, 169.8 (C = O), 177.6 (Ar-C = O). FT-IR (KBr disc, ν cm-1); 3088, 3066 (Csp2-H, benzene ring stretch) 2850, 2939, 2974 (C_sp_^3^-H, alkyl groups), 1644, 1740 (C = O, carbonyl groups), 1604, 1574 (C = C stretch aromatic), 1070, 1094 (alkoxy C-O stretch), 904 and 920 cm^-1^. MS (m/z, M^+^) for C_23_H_20_O_6_ calcd 392.11 and found 393.1. The characteristic features of 2D ^1^H-^1^H COSY, HSQC and HMBC experiments were found comparable to the previous reports (Refer to Figures S1 to S6 in File S1) [11], [22].

### Cell Lines and Culture Conditions

Nine cancer cell lines and one normal cell line were used in this study: Human colorectal carcinoma cell line HCT-116; catalogue number (CCL-247); human hormone sensitive and invasive breast cancer cell line MCF-7; catalogue number (HTB-22); human colorectal normal cell line CCD-18; catalogue (CRL-1459); prostate cancer cell line PC3 catalogue number (46652); human leukemia Cell line HL-60 (CCL-240); K562 catalogue number (CCL-243); U937 catalogue number (40759), HT-29 catalogue number (HTB-38) and human hepatic carcinoma (HepG2) cells; catalogue (CRL-10741) were purchased from ScienCell, USA. HCT-116, HT-29, HL-60, K562, U937 and HepG2 cells were maintained in RPMI; MCF-7 and RGC-5 were maintained in DMEM and PC3 was maintained in F-12K medium. The media were supplemented with 5% heat inactivated fetal bovine serum and 1% penicillin/streptomycin. Cells were cultured in a humidified incubator at 37°C supplied by 5% CO_2_.

### *In Vitro* Cytotoxic Assay

Cytotoxicity of SSG was evaluated using MTT assay [47], [50] against a panel of cell lines. The assay plates were read using a microtiter plate reader (Hitachi U-2000, Japan) at 570 nm absorbance. DMSO (0.1%) was used as a negative control. Tamoxifen, betulinic acid, 5-fluorouracil and imatinib were used as standard references.

### Determination of Nuclear Condensation by Hoechst 33342 Stain

Effect of SSG on nuclear chromatin condensation in PC3, MCF7 and HCT 116 cells was quantified by fluorescence microscopy using Hoechst 33258 stain [51]. Cells were treated with SSG (10 µM) and analysed separately at two different time intervals (6 and 18 hr). Betulinic acid (10 µM) and 0.1% DMSO were used as positive and negative controls, respectively. The cells were fixed in 4% paraformaldehyde for 20 min before staining with Hoechst stain 33342 (1 µg/mL in PBS) for 20 min. Nuclear condensation and cytoplasm shrinkage was examined under a fluorescent microscope. Cells with bright colored, condensed or fragmented nuclei were considered apoptotic. The number of cells with apoptotic morphology was counted in randomly selected fields per well. The cells were photographed at 20× magnification, using a EVOS f1 digital microscope (Advanced Microscopy Group, USA). The apoptotic index was calculated as a percentage of apoptotic nuclei compared to the total number of cells and presented as the mean ± SD (n  =  8).

### Detection of Mitochondrial Membrane Potential by Rhodamin 123 Stain

Detection of the changes in mitochondrial membrane potential in PC3 cells treated with SSG was assessed by the retention of rhodamine 123 [26]. The PC3 cells were plated in 6 well plates for overnight. The cells were treated with SSG at 10 µM for 6 and 18 hr intervals and then fixed by 4% paraformaldehyde for 20 min. Betulinic acid (10 µM) and 0.1% DMSO were used as positive and negative controls, respectively. The rhodamine 123 was added to cells at a final concentration of 5 µg/mL and incubated for 30 min to stain the mitochondria. The wells then were photographed using inverted EVOS f1 digital microscope at 20× magnification power to monitor for fluorescent signals.

### Statistical Analysis

Statistical difference between the treatments and the control were evaluated by one-way analysis of variance (ANOVA) followed by Tukey's multiple comparison test. Differences were considered significant at p<0.05, and p<0.01.

## Conclusions

In the present work, the isolation of (-)-pseudosemiglabrin (SSG) from aerial parts of *T. apollinea* and its detailed stereochemistry and antiproliferative activity is reported. From the results, it can be concluded that SSG has strong cytotoxic properties selectively against prostate, leukemia, breast and colon cancer cells. Eventually the pro-apoptotic property could be the principle factor for the observed cytotoxicity of SSG. Further, the *in vivo* antitumor studies of title compound are in progress using Ncr-nu/nu mice xenograft models and will be reported in due course.

## Additional Information

Crystallographic data of the structures have been deposited with the Cambridge Crystallographic Data Center, CCDC 946618 for (-)-pseudosemiglabrin. This data can be obtained free of charge from CCDC via www.ccdc.cam.ac.uk/data_request/cif.

## Supporting Information

File S1 NMR Spectral and crystal data of (-)-pseudosemiglabrin. **Figure S1** depicts the 13C DEPT135 NMR spectrum of (-)-pseudosemiglabrin collected in CDCl3 at ambient temperature (125.7 MHz). **Figure S2** shows the 13C DEPT145 NMR spectrum of (-)-pseudosemiglabrin collected in CDCl3 at ambient temperature (125.7 MHz). **Figure S3** illustrates the 2D HSQC (Heteronuclear single quantum coherence spectroscopy) NMR spectrum of (-)-pseudosemiglabrin collected in CDCl3 at ambient temperature. **Figure S4** shows 2D HMBC (heteronuclear multiple-bond correlation spectroscopy) NMR spectrum of (-)-pseudosemiglabrin collected in CDCl3 at ambient temperature. **Figure S5** depicts the 2D TOCSY (total correlation spectroscopy) NMR spectrum of (-)-pseudosemiglabrin collected in CDCl3 at ambient temperature. **Figure S6** shows the 2D COSY (correlation spectroscopy) NMR spectrum of (-)-pseudosemiglabrin collected in CDCl3 at ambient temperature. **Figure S7** illustrates the crystal packing of (-)-Pseudosemiglabrin. The molecules packed in orthorhombic crystal system through intermolecular hydrogen bonds (C = O---H), shown as dashed lines. **Table S1** describes the selected Bond Lengths (Å) and Angles (o) of (-)-Pseudosemiglabrin (Crystal Structure Unit A). **Table S2** describes the selected Bond Lengths (Å) and Angles (o) of (-)-Pseudosemiglabrin (Crystal Structure Unit B).
(PDF)
