# Supplementary material for: Crystal Structure Elucidation and Anticancer Studies of (-)-Pseudosemiglabrin: A Flavanone Isolated from the Aerial Parts of Tephrosia apollinea
Source: PLoS One. 2014 Mar 7;9(3):e90806. doi: 10.1371/journal.pone.0090806 (PMC3946547; doi:10.1371/journal.pone.0090806)
Supplement: File S1 — NMR Spectral and crystal data of (-)-pseudosemiglabrin. Figure S1 depicts the 13C DEPT135 NMR spectrum of (-)-pseudosemiglabrin collected in CDCl3 at ambient temperature (125.7 MHz). Figure S2 shows the 13C DEPT145 NMR spectrum of (-)-pseudosemiglabrin collected in CDCl3 at ambient temperature (125.7 MHz). Figure S3 illustrates the 2D HSQC (Heteronuclear single quantum coherence spectroscopy) NMR spectrum of (-)-pseudosemiglabrin collected in CDCl3 at ambient temperature. Figure S4 shows 2D HMBC (heteronuclear multiple-bond correlation spectroscopy) NMR spectrum of (-)-pseudosemiglabrin collected in CDCl3 at ambient temperature. Figure S5 depicts the 2D TOCSY (total correlation spectroscopy) NMR spectrum of (-)-pseudosemiglabrin collected in CDCl3 at ambient temperature. Figure S6 shows the 2D COSY (correlation spectroscopy) NMR spectrum of (-)-pseudosemiglabrin collected in CDCl3 at ambient temperature. Figure S7 illustrates the crystal packing of (-)-Pseudosemiglabrin. The molecules packed in orthorhombic crystal system through intermolecular hydrogen bonds (C = O---H), shown as dashed lines. Table S1 describes the selected Bond Lengths (Å) and Angles (o) of (-)-Pseudosemiglabrin (Crystal Structure Unit A). Table S2 describes the selected Bond Lengths (Å) and Angles (o) of (-)-Pseudosemiglabrin (Crystal Structure Unit B). (PDF) [file pone.0090806.s001.pdf]

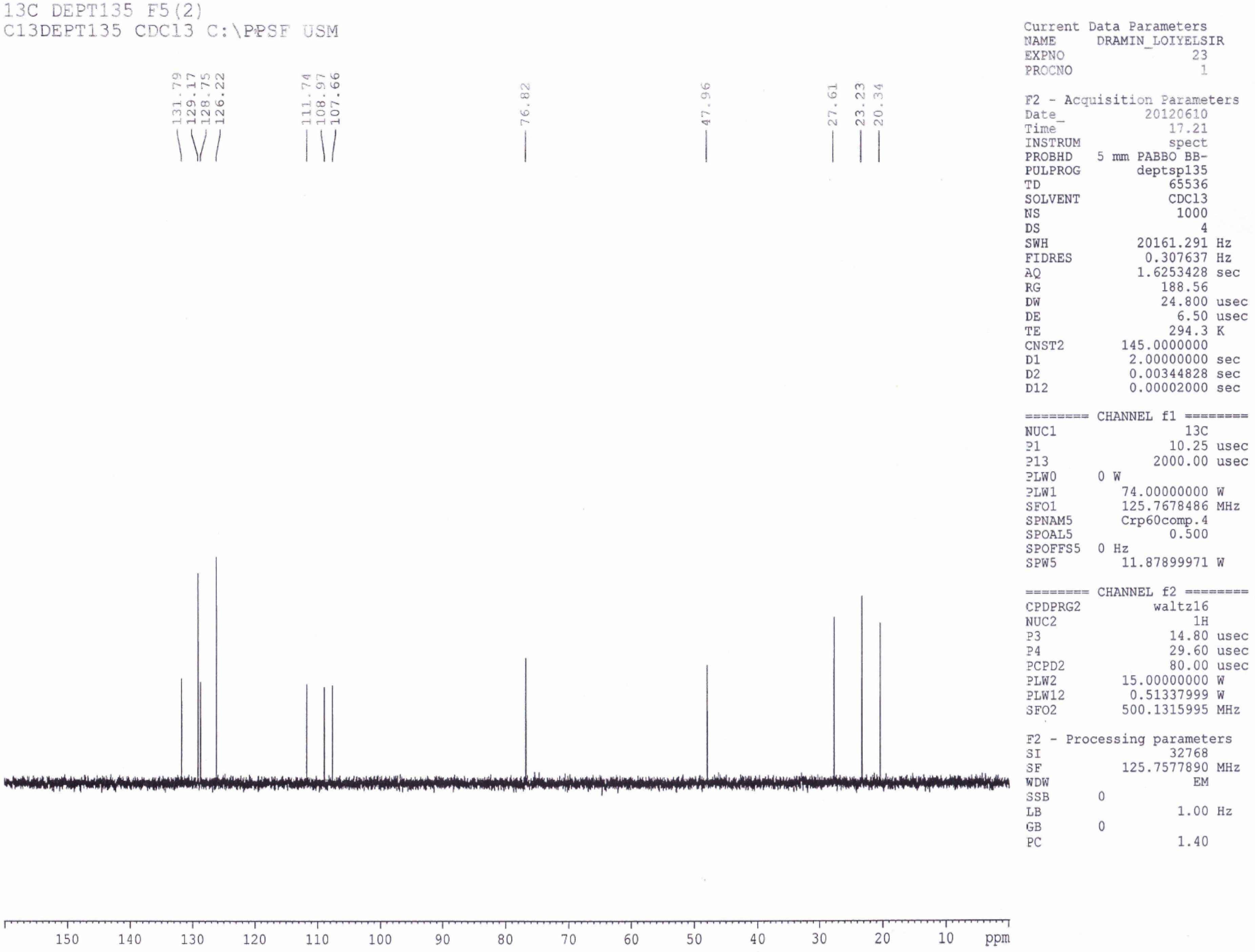

Figure S1: **13C DEPT135 NMR spectrum of (-)-pseudosemiglabrin collected in CDCl3 at ambient temperature (125.7 MHz).**

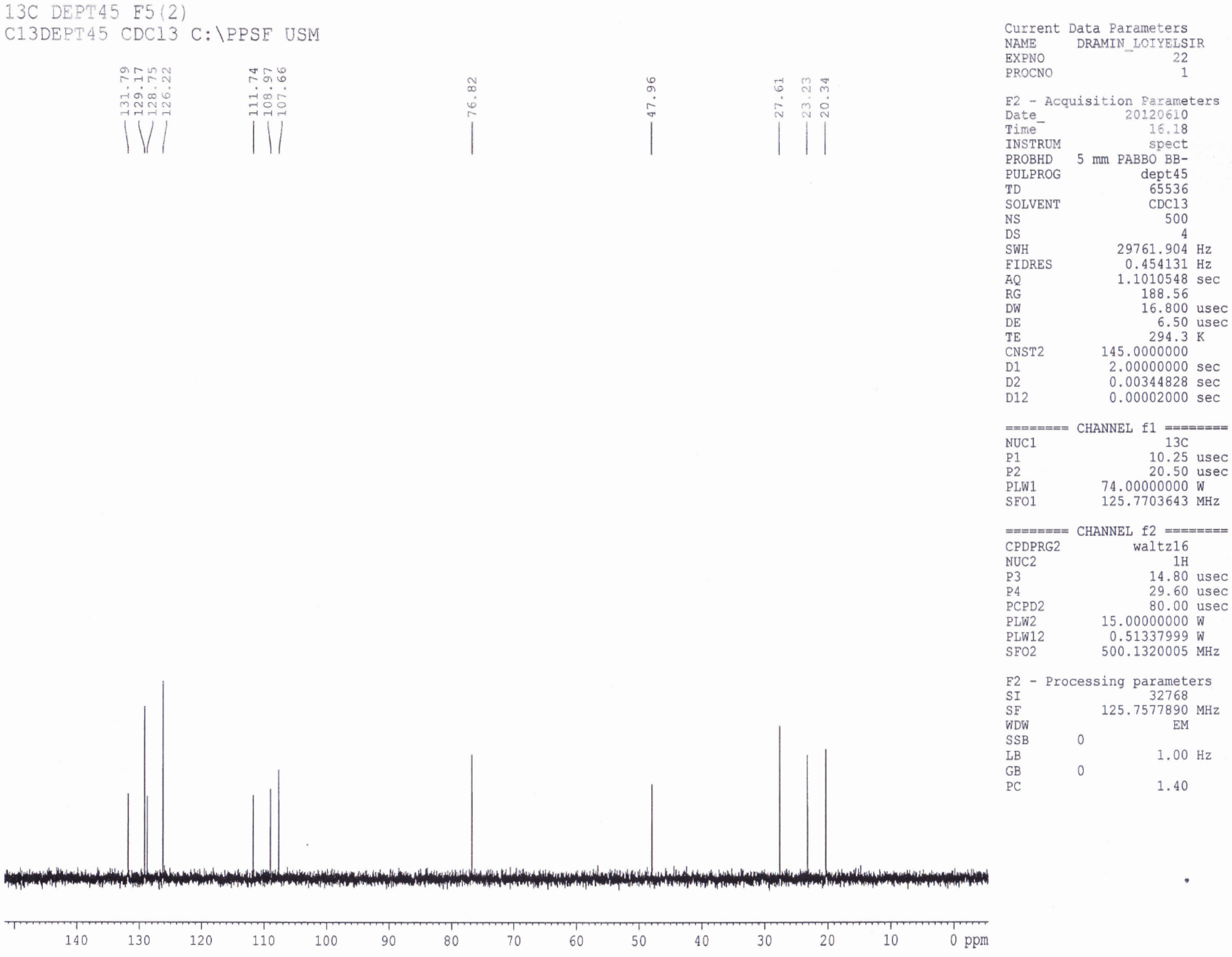

Figure S2: **13C DEPT145 NMR spectrum of (-)-pseudosemiglabrin collected in CDCl3 at ambient temperature (125.7 MHz).**

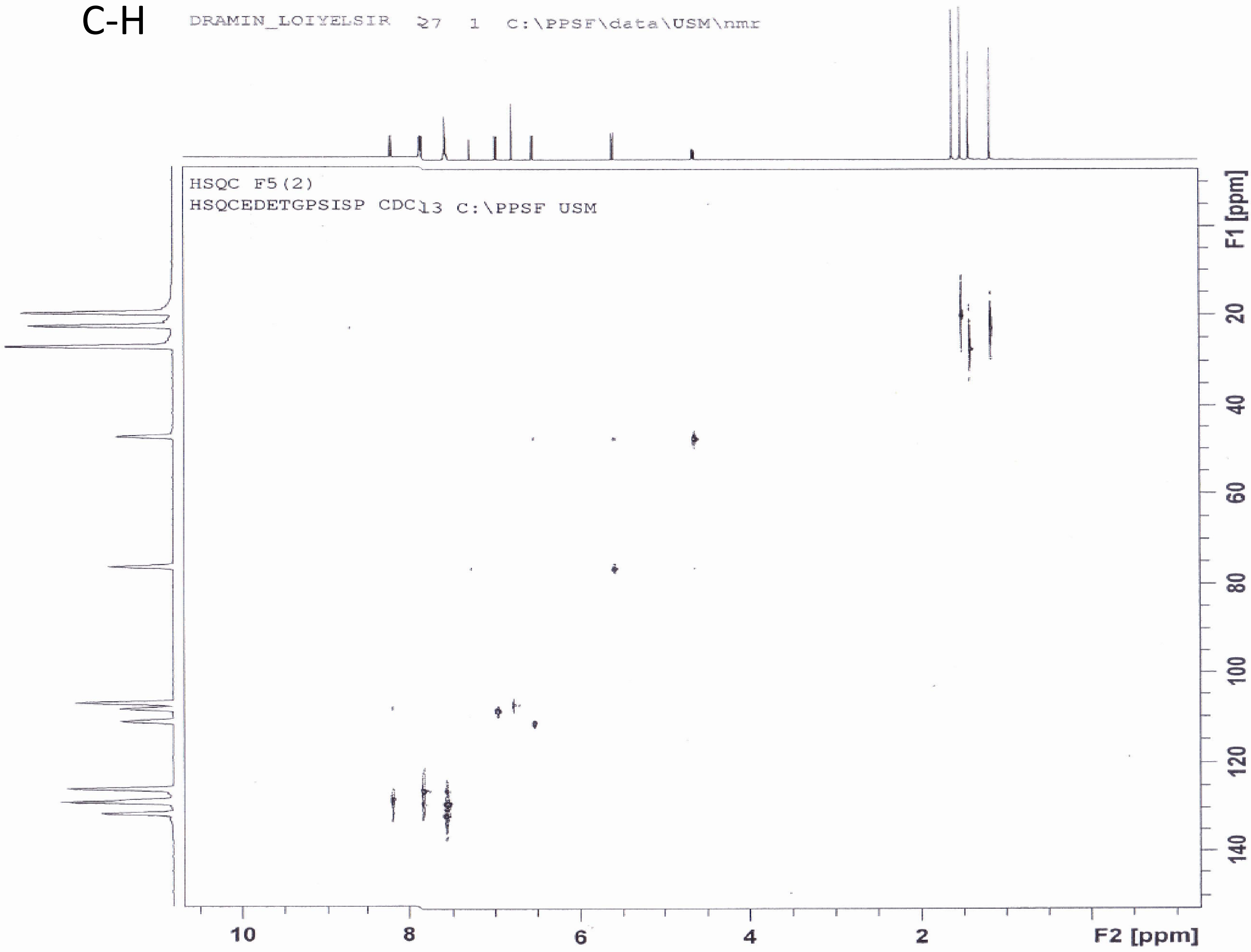

Figure S3: **2D HSQC (Heteronuclear single quantum coherence spectroscopy) NMR spectrum of (-)-pseudosemiglabrin collected in CDCl3 at ambient temperature.**

C-(-)$_n$ H

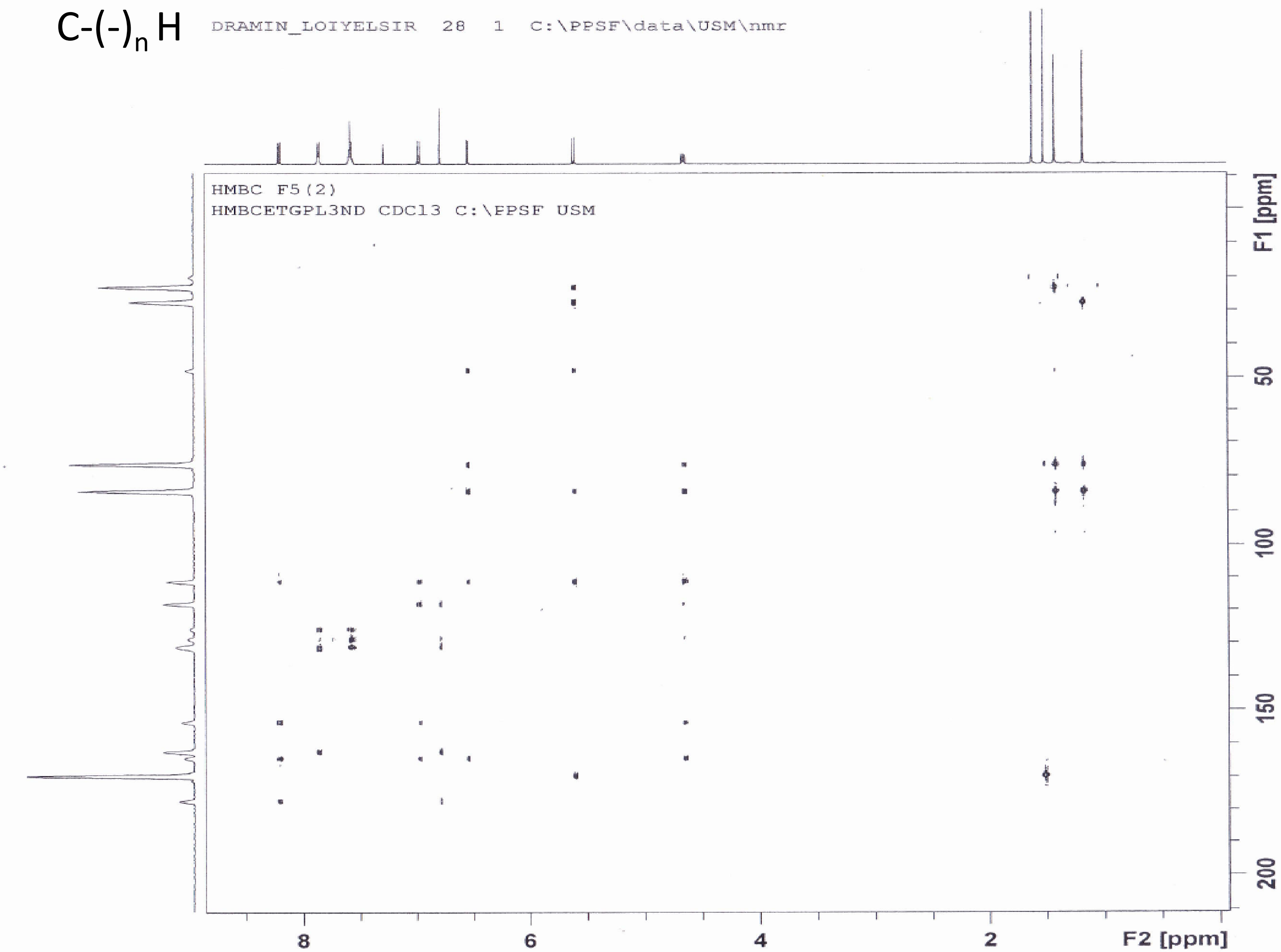

Figure S4: 2D HMBC (heteronuclear multiple-bond correlation spectroscopy) NMR spectrum of (-)-pseudosemiglabrin collected in CDCl3 at ambient temperature.

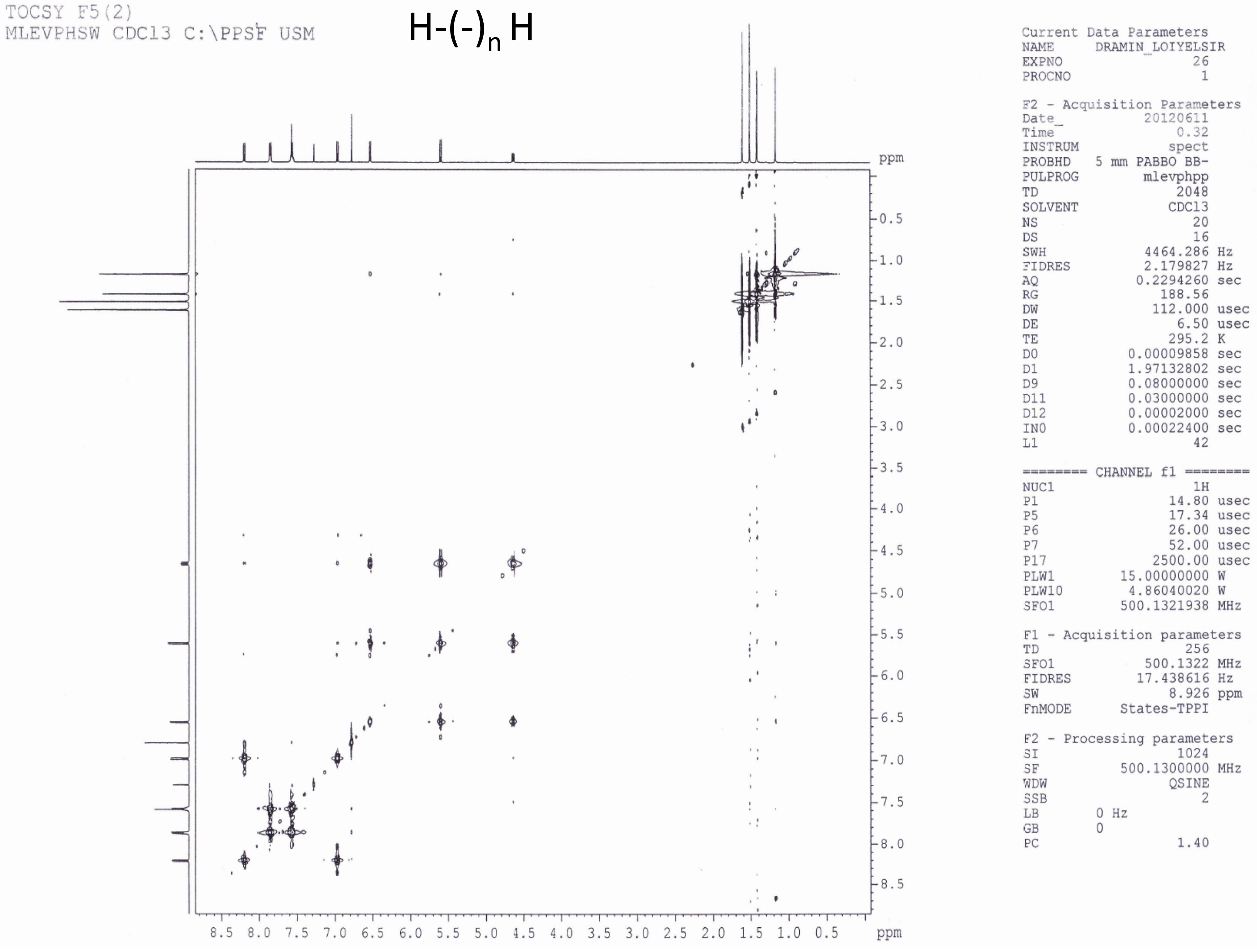

Figure S5: **2D TOCSY (total correlation spectroscopy) NMR spectrum of (-)-pseudosemiglabrin collected in CDCl3 at ambient temperature.**

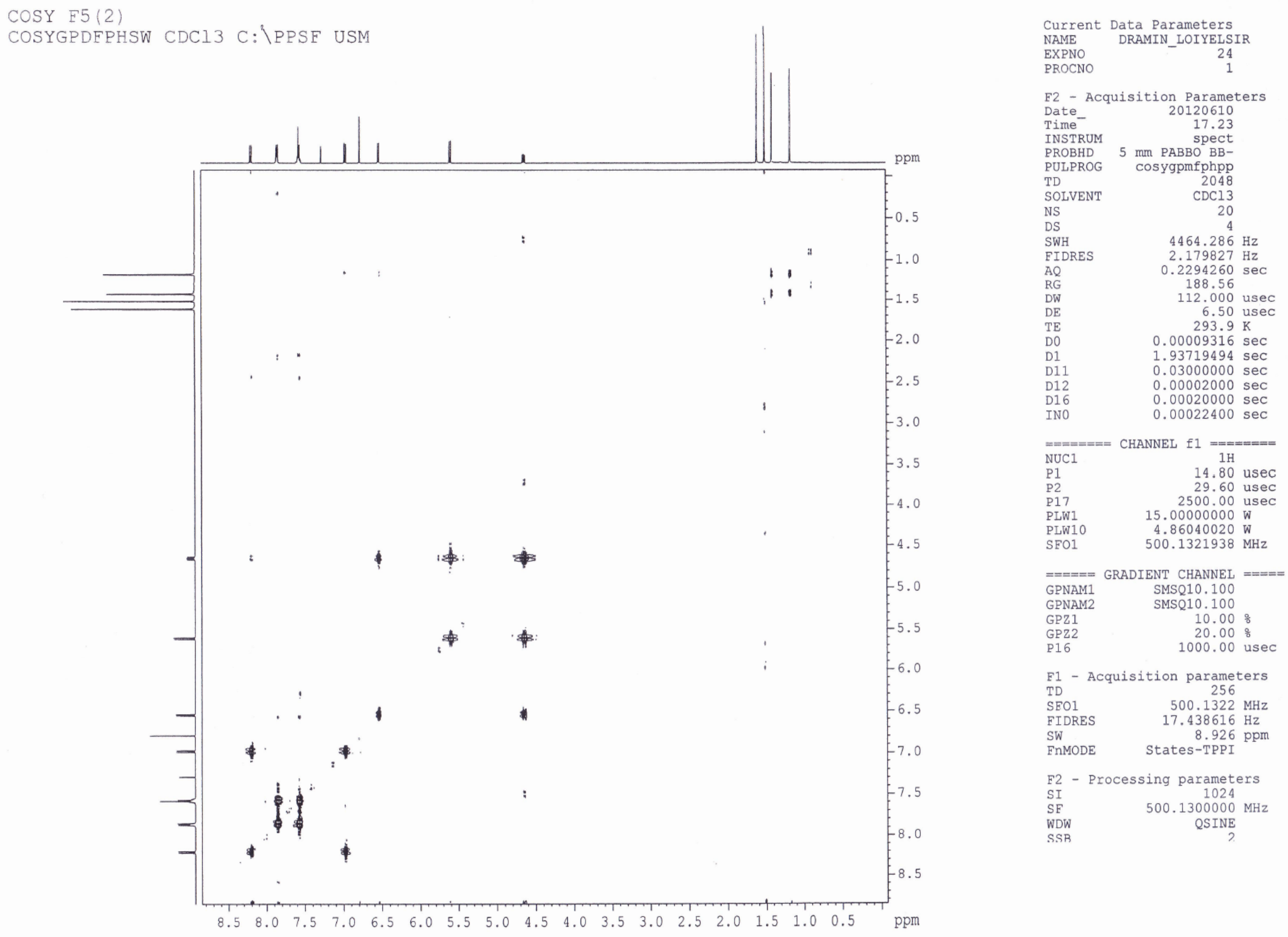

Figure S6: **2D COSY (correlation spectroscopy) NMR spectrum of (-)-pseudosemiglabrin collected in CDCl3 at ambient temperature.**

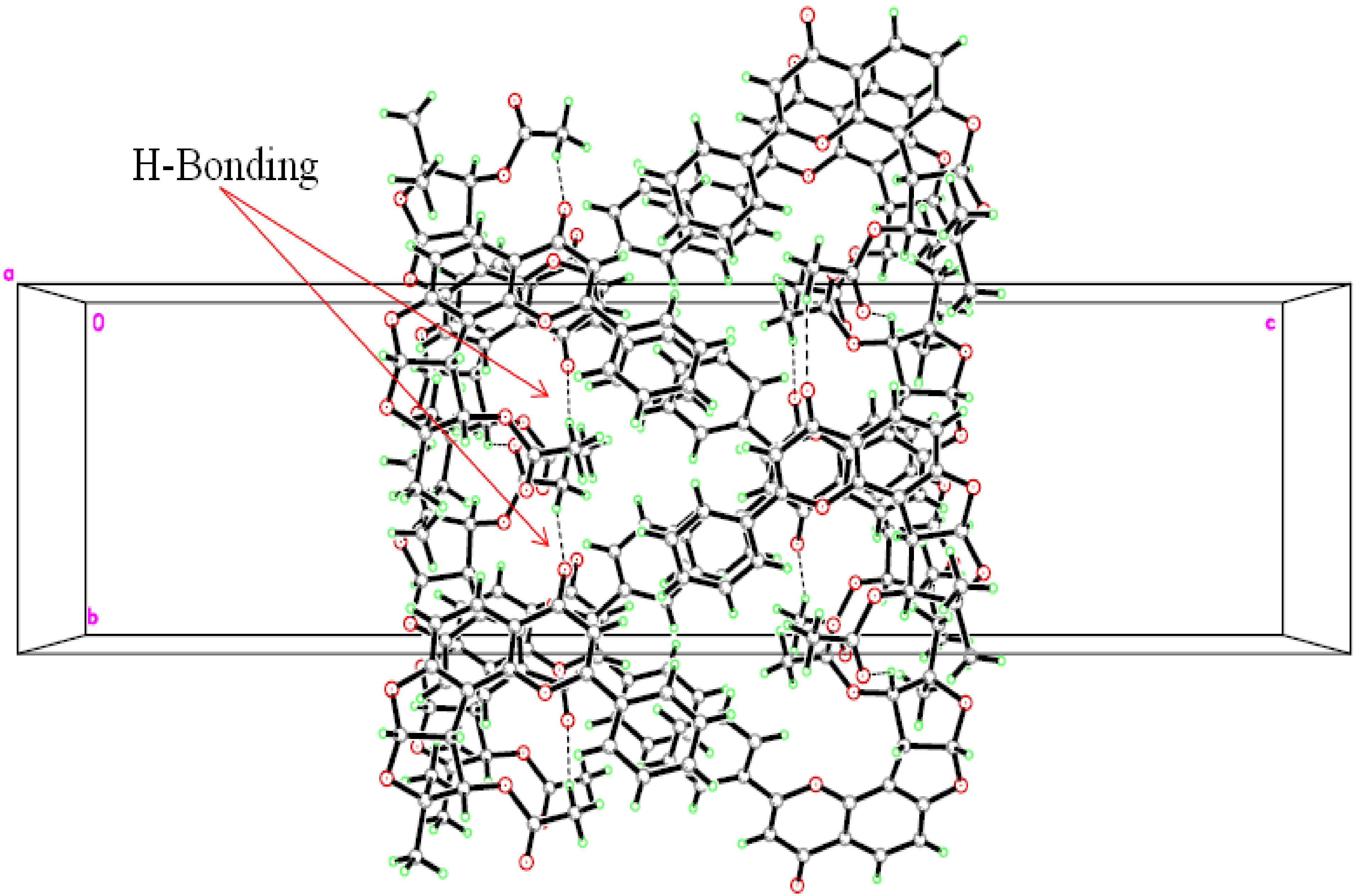

Figure S7: **Crystal packing of (-)-Pseudosemiglabrin. The molecules packed in orthorhombic crystal system through intermolecular hydrogen bonds (C=O---H), shown as dashed lines.**

Table S1: Selected Bond Lengths (Å) and Angles (º) of (-)-Pseudosemiglabrin

(Crystal Structure Unit A).

C15A-O3A	1.461(2)	C9A-05A	1.237(3)	C17A-C18A	1.505(3)
O3A-C14A	1.393(2)	C22A-O6A	1.197(3)	C14A-C17A	1.551(3)
C14A-O2A	1.461(2)	C7A-C6A	1.462(12)		
O2A-C13A	1.369(2)	C6A-C5A	1.475(3)		
C19A-O1A	1.370(2)	C6A-C1A	1.402(3)		
O1A-C7A	1.367(2)	C16A-C17A	1.539(3)		
O2A-C14A-O3A	110.73(15)		C14A-O2A-C13A	108.27(14)	
C18A-C17A-C16A	116.54(16)		C15A-O3A-C14A	110.58(14)	
C21A-C15A-C20A	110.57(16)		C19A-O1A-C7A	118.93(15)	
C13A-C12A-C11A	116.49(18)		C16A-O4A-C22A	117.41(15)	
C10A-C9A-C8A	114.25(17)		O4A-C22A-C23A	110.29(16)	
C1A-C6A-C5A	118.72(18)				

Table S2: Selected Bond Lengths (Å) and Angles (º) of (-)-Pseudosemiglabrin

(Crystal Structure Unit B).

C15B-O3B	1.456(2)	C9B-05B	1.237(2)	C17B-C18B	1.504(3)
O3B-C14B	1.392(2)	C22B-O6B	1.202(3)	C14B-C17B	1.552(3)
C14B-O2B	1.471(2)	C7B-C6B	1.472(3)		
O2B-C13	1.364(2)	C6B-C5B	1.403(3)		
C19B-O1B	1.373(2)	C6B-C1B	1.396(3)		
O1B-C7B	1.368(2)	C16B-C17B	1.549(3)		
O2B-C14B-O3B	110.58(15)		C14B-O2B-C13B	108.53(14)	
C18B-C17B-C16B	116.18(16)		C15B-O3B-C14B	111.24(14)	
C21B-C15B-C20B	111.06(16)		C19B-O1B-C7B	118.96(15)	
C13B-C12B-C11B	116.63(18)		C16B-O4B-C22B	117.07(15)	
C10B-C9B-C8B	114.32(17)		O4B-C22B-C23B	110.94(16)	
C1B-C6B-C5B	118.67(18)				
